# Supplementary material for: Mathematical and statistical approaches in epidemiological investigation of hospital infection: A case study of the 2015 Middle East Respiratory Syndrome outbreak in Korea
Source: PLoS One. 2025 Jan 24;20(1):e0317083. doi: 10.1371/journal.pone.0317083 (PMC11759389; doi:10.1371/journal.pone.0317083)
Supplement: Attachment — Submitted filename: Reviewer report_PONE-D-24-28130.docx [file pone.0317083.s001.docx]

**Reviewer report**

**Manuscript Number**: PONE-D-24-28130

**Title**: Mathematical and Statistical Approaches in Epidemiological Investigation of Hospital Infection: A Case Study of the 2015 Middle East Respiratory Syndrome Outbreak in Korea

**Summary:**

Much as the authors address an important diseases, they need to improve in their writing style to improve clarity. There is also an element of plagiarism detected and this needs to be avoided.

**Some of the Major observations:**

1. The abstract as currently written does not have easily identifiable sections. The flow of content should be clear enough that the reader can identify the introduction, materials and methods, results and conclusion. For example, there is no introduction at all and the sentences can also be improved upon in general. Generally rewrite the abstract.
2. There is a lot of copy-paste verbatim detected, moreover without citation. For example in lines 4-5 you write “The primary purpose of these investigations is to identify the occurrence of an outbreak or unusual increase in number of cases.”. This is the exact text as written in <https://www.studysmarter.co.uk/explanations/medicine/epidemiology/outbreak-investigation/>
3. Improve clarity of most of your sentences. For example, in lines 7-8 you write “These data are then used to implement control measures to prevent further spread of the disease and to forestall future outbreaks.” However, it is not clear how data alone can be used to achieve that. Rather, the data is used to inform studies geared towards assessing the effectiveness of control measures.
4. In general, the flow of materials and methods needs to be heavily improved upon. For example, in lines 42-45, it is not clear how the two listed factors influence the probability of infection. I think the intended message is totally lost in the phrasing of the sentences.
5. There is need to cite relevant literature throughout. For example, the derivations in equations (3)-(5) have already been published in several articles for example: 1) Dietz K and Schenzle D (1985) Proportionate mixing models for age-dependent infection transmission. Journal of Mathematical Biology 22, 117–120. 2) Becker NG (1989) Analysis of Infectious Disease Data. London: Chapman and Hall. And 3) Ssematimba A et al. (2018) Estimating the between-farm transmission rates for highly pathogenic avian influenza subtype H5N1 epidemics in Bangladesh between 2007 and 2013. Transboundary and Emerging Diseases 65, e127–e134. The authors need to acknowledge that.
6. You are categorize events as Markovian and non-Markovian processes in the model events. This is based on the “memoryless property” of events. You may need to clarify/justify your choices for each event for the benefit of the readers.

**Some of the minor observations:**

1. In the abstract address the typographic error in “…by a maximum of 77Through…” There is also a missing “%” and minimum percentage.
2. In line 17: You write “… age groups during COVID-19,…” add pandemic for completeness.
3. Line 80: Use “indicating”
4. There is a typo in Equation (6) change L_s to L_i in the second likelihood function.
5. In line 99: mention the exact MATLAB function that you are referring to.
6. In line 107, 123, 126, 153, 166 and elsewhere: spell out the “d” in “…PMH for 3 d…”?
7. In the results section you use “Confidence Interval” and “Credibility Interval”. One is frequentist and the other is Bayesian. Be sure that you are rightly doing so based on the methods used in each case.
8. Line 149: You write “…cases was 36.12, which was close to the actual number of cases…” For ease of comparison, mention here the actual number of cases.
9. In line 188: what do you mean be “in order of”? Consider rephrasing the sentence for improved clarity.
10. Generally improve on your sentence to enhance the flow of content
