# Supplementary material for: Mathematical and statistical approaches in epidemiological investigation of hospital infection: A case study of the 2015 Middle East Respiratory Syndrome outbreak in Korea
Source: PLoS One. 2025 Jan 24;20(1):e0317083. doi: 10.1371/journal.pone.0317083 (PMC11759389; doi:10.1371/journal.pone.0317083)
Supplement: Attachment — Submitted filename: Reviewers comments.docx [file pone.0317083.s002.docx]

**Reviewer #1:**
This is an interesting study and it enhances the existing knowledge base on modeling and parameter estimation. However, you need improve on the writing style for better content flow. Also always avoid copy-paste verbatim of content.

**Answer**

We revised the paper to improve readability and avoid any potential controversy. Following are example revised parts:

Abstract: We revised the abstract section as follows for better readability and structure:

*Mathematical and statistical methods are invaluable in epidemiological investigations, enhancing our understanding of disease transmission dynamics and informing effective control measures. In this study, we presented a method to estimate transmissibility using patient-level data, with application to the 2015 MERS outbreak at Pyeongtaek St. Mary’s Hospital, the Republic of Korea. We developed a stochastic model based on individual case data to derive a likelihood function for disease transmission. Through scenario-based analysis, we explored transmission dynamics, including the role of superspreaders, and investigated how mask-wearing impacted infection control within the hospital. Our findings indicated that the superspreader during the MERS outbreak had approximately 25 times higher transmissibility compared to other patients. Under scenarios of prolonged hospital transmission periods, the number of cases could potentially increase threefold. The impact of mask-wearing in the hospital was significant, with reductions in the epidemic scale ranging from 17\% to 77\%, depending on the type of mask and intervention intensity. This study quantifies key risk factors in hospital-based transmission, demonstrating the effectiveness of intervention measures. The methodology developed here can be readily adapted to other infectious diseases, providing valuable insights for future outbreak preparedness and response strategies.*

In introduction: L2-8

*An epidemiological investigation is a systematic method used to determine the cause, source, and spread of a disease within a population, usually following an unusual increase in the number of cases. This process typically involves steps like verifying the outbreak, defining and identifying additional cases, using epidemiological methods to understand the disease transmission dynamics, and collecting data pertaining to cases, their characteristics, and potential risk factors.These data are directly utilized to estimate model parameters and simulate realistic outbreak patterns. This allows for evidence-based implementation of control measures to prevent further spread of the disease and reduce the risk of future outbreaks.*

L13-17

*For instance, during the COVID-19 pandemic in South Korea, detailed epidemiological information on individual cases—including estimated exposure dates, symptom onset dates, and reporting dates—was collected and analyzed using mathematical and statistical methods until the Omicron variant became dominant.*

**Reviewer #2:**

**Comment #1**
Line 14-16: “For example, during the Coronavirus disease 2019 (COVID-19) pandemic… [8-11]”. Does the statement refer to the COVID-19 pandemic worldwide or only isolated to Korea? In the four cited references there are studies only from Korea.

**Answer #1**

This section was included to introduce an example of large-scale collection and analysis of individual case information during the COVID-19 pandemic. We revised it as follows (L13-17):

*For instance, during the COVID-19 pandemic in South Korea, detailed epidemiological information on individual cases—including estimated exposure dates, symptom onset dates, and reporting dates—was collected and analyzed using mathematical and statistical methods until the Omicron variant became dominant.*

========================================================================

**Comment #2**
Line 19-21: “By integrating epidemiological investigations… of model results”. Is this an assumption or a statement? If it is a statement, please add the reference.

**Answer #2**

We cited two new papers, along with four additional papers referenced in the previous sentence:

- Shim E, Choi W, Song Y. Clinical time delay distributions of COVID-19 in 2020–2022 in the Republic of Korea: inferences from a nationwide database analysis. J Clin Med. 2022;11(12):3269.
- Ko Y, Lee J, Seo Y, Jung E. Risk of COVID-19 transmission in heterogeneous age groups and effective vaccination strategy in Korea: a mathematical modeling study. Epidemiology and Health. 2021;43.
- Ko Y, Lee J, Kim Y, Kwon D, Jung E. COVID-19 vaccine priority strategy using a heterogenous transmission model based on maximum likelihood estimation in the Republic of Korea. Int J Environ Res Public Health. 2021;18(12):6469.
- Ko Y, Mendoza VMP, Seo Y, Lee J, Kim Y, Kwon D, et al. Quantifying the effects of non-pharmaceutical and pharmaceutical interventions against Covid-19 epidemic in the Republic of Korea: mathematical model-based Approach considering age groups and the delta variant. Math Model Nat Phenom. 2022;17:39.
- Anderson RM, May RM. Infectious diseases of humans: dynamics and control. Oxford university press; 1991.
- Hazelbag CM, Dushoff J, Dominic EM, Mthombothi ZE, Delva W. Calibration of individual-based models to epidemiological data: A systematic review. PLoS computational biology. 2020 May 11;16(5):e1007893.

**Comment #3**
Line 24-27: “As a case study, we apply this method to an outbreak at Pyeongtaek… where 36 out of 186 total cases were identified [12–14]”. According to reference 12, the 186 cases (36 death and 138 recovered cases) were reported until July 26, 2015. According to figure 2 from reference 12 "Distribution of transmission of Middle East respiratory syndrome coronavirus clusters and suspected super spreaders in South Korea (20th May to 25th November 2015)" the epidemiological analysis was carried out. What is the new information presented in your study?

**Answer #3**

In this study, we presented insights into estimating transmission rates within the context of a specific outbreak at Pyeongtaek, utilizing general mathematical and statistical methods tailored for epidemiological investigations. We addressed the heterogeneity of hosts in a hospital environment, categorizing individuals into superspreaders, healthcare workers, patients, and visitors. This allowed us to develop a model that accurately reflects the dynamics of the outbreak and simulates realistic transmission scenarios. We also demonstrated that this method could be easily applied elsewhere. To enhance readability, we moved the section discussing “estimating transmission rate” forward and revised the sentences accordingly (L32-42).

*To investigate transmission rates in detail, we categorized individuals into superspreaders, healthcare workers (HCWs), patients, and visitors based on actual epidemiological investigations. The spread in Korea was largely due to superspreaders, patients who caused secondary infections in more than six people. Of the 186 cases, it was suspected that 15 transmitted the disease to others, with five identified as superspreaders.\\*

*Simultaneously with the estimation of the transmission rate, we developed a model that reflects these investigations and simulates a realistic outbreak.A scenario-based analysis was introduced to quantify risk factors such as the infectious period of infected individuals, including superspreaders, and the effectiveness of mask mandates within the hospital.*

**Comment #4**
Line 27-30: “The spread in Korea… than six people [15]. Of the 186 cases, it was suspected that 15 transmitted the disease to others, with five identified as superspreaders.” The "super-spread" terminology used in the text was taken from reference 15. Please add the appropriate reference.

**Answer #4**

After reviewing the content, we added the following references. These studies include definitions of superspreaders, associated risks, and cases from Korea:

[Additional references]

- Lloyd-Smith JO, Schreiber SJ, Kopp PE, Getz WM. Superspreading and the effect of individual variation on disease emergence. Nature. 2005 Nov 17;438(7066):355-9.\\
- Cho SY, Kang JM, Ha YE, Park GE, Lee JY, Ko JH, Lee JY, Kim JM, Kang CI, Jo IJ, Ryu JG. MERS-CoV outbreak following a single patient exposure in an emergency room in South Korea: an epidemiological outbreak study. The Lancet. 2016 Sep 3;388(10048):994-1001.\\
- Park JE, Jung S, Kim A, Park JE. MERS transmission and risk factors: a systematic review. BMC public health. 2018 Dec;18:1-5.

=============================================================================

**Comment #5**
Line 36-38: “The methodology used in this study, which relatively easily transforms the situation of hospital infections into a likelihood function, can be applied to other hospitals or infectious disease outbreaks.” Is this sentence an assumption or is it demonstrated by other articles that use this method for other infectious diseases?

**Answer #5**

In response to the comment, we revised it for clarity. This revision emphasizes that the methodology is not merely an assumption but is supported by its successful application in various contexts, thereby demonstrating its relevance and effectiveness in estimating transmission rates in different infectious disease scenarios. The updated version (L42-49) states:

*The methodology employed in this study transforms the dynamics of hospital infections into a likelihood function, allowing for its application to other hospitals or infectious disease outbreaks. By focusing on the estimation of transmission rates among heterogeneous hosts in certain spaces like hospitals, we expect these findings to significantly enhance both reactive and preventive measures. This approach, which has been successfully utilized in various contexts, including human-to-human and animal-to-animal infectious diseases, underscores the potential for improving infection control strategies*

[Additional references]

- Keeling MJ, Woolhouse ME, Shaw DJ, Matthews L, Chase-Topping M, Haydon DT, Cornell SJ, Kappey J, Wilesmith J, Grenfell BT. Dynamics of the 2001 UK foot and mouth epidemic: stochastic dispersal in a heterogeneous landscape. Science. 2001 Oct 26;294(5543):813-7.
- Boender GJ, Hagenaars TJ, Bouma A, Nodelijk G, Elbers AR, de Jong MC, Van Boven M. Risk maps for the spread of highly pathogenic avian influenza in poultry. PLoS computational biology. 2007 Apr;3(4):e71.

**Comment #6**
Line 230-234: “The overall methodology of this study can be applied to other outbreak situations… to the specific conditions in future outbreak situations.” Considering that the present study was submitted in 2024, after the WHO declared that the COVID-19 pandemia had ended, I would have liked you to compare MERS and SARS-CoV-2 in the perspective in which the methodology used can be inter-connected.

**Answer #6**

In the Republic of Korea, during the early stages of the COVID-19 pandemic, there were several reports of outbreaks in closed environments such as hospitals, nursing homes, and correctional facilities. If we can obtain individual case data specific to each location, it seems feasible to apply this methodology. Taking this comment into account, I will aim to conduct comparative studies in the future when we have processed data categorized by space. We added following sentence in discussion section (L253-259)

*Additionally, our methodology could be adapted to other outbreaks in closed settings, such as those documented early in the COVID-19 pandemic in the Republic of Korea, in environments like hospitals, nursing homes, and correctional facilities. With access to individual case data specific to each location, we believe our approach could be effectively applied to analyze similar scenarios. Furthermore, as recorded in endemic regions, future studies will highlight the transmission pathways moving from environmental sources to humans and subsequently to healthcare facilities.*

[Added references]

- Lee HA, Ahn MH, Byun S, Lee HK, Kweon YS, Chung S, Shin YW, Lee KU. How COVID-19 affected healthcare workers in the hospital locked down due to early COVID-19 cases in Korea. Journal of Korean Medical Science. 2021 Dec 6;36(47).
- Giri S, Chenn LM, Romero-Ortuno R. Nursing homes during the COVID-19 pandemic: a scoping review of challenges and responses. European Geriatric Medicine. 2021 Dec;12(6):1127-36.
- Lee HY, Park YJ, Yu M, Park H, Lee JJ, Choi J, Park HS, Kim JY, Moon JY, Lee SE. Accuracy of rapid antigen screening tests for SARS-CoV-2 infection at correctional facilities in Korea: March-May 2022. Infection \& Chemotherapy. 2023 Dec;55(4):460.

**Reviewer #3:**

**Comment #1**
It is well known that the MERS outbreak in South Korea was an explosive outbreak in healthcare settings which needs more demonstrated in the text and added in the model. The outbreak largely relied on the patient's health-seeking behaviors, so it should be reflected in their model.

**Answer #1**

In the actual 2015 MERS outbreak, health-seeking behaviors (also referred to as “doctor shopping”) contributed to the spread across multiple hospitals. In our study, however, we limited the model to a single hospital (Pyeongtaek St. Mary's Hospital) and did not consider this behavior. In reality, case #14, responsible for further spread, was initially infected at Pyeongtaek and subsequently admitted to a larger hospital (Samsung Medical Center), where subsequent transmissions occurred. We believe that health-seeking behaviors could be effectively addressed in an advanced model that considers both hospitals and the community. To address this comment, we have added the following statement in the Discussion section (L239-259):

*In this study, we focused on a single hospital context and did not include health-seeking behaviors—such as visiting multiple healthcare facilities, often called 'doctor shopping'—which significantly contributed to the cross-hospital spread during the 2015 MERS outbreak in the Republic of Korea. Addressing this and other complexities, such as detailed spatial arrangements, individual patient tracking, isolation and testing procedures, and infections involving HCWs, would allow for a more comprehensive model. Future work will expand on these elements, incorporating diverse transmission types and behaviors to better capture the dynamics of healthcare-associated outbreaks.*

*Additionally, our methodology could be adapted to other outbreaks in closed settings, such as those documented early in the COVID-19 pandemic in the Republic of Korea, in environments like hospitals, nursing homes, and correctional facilities. With access to individual case data specific to each location, we believe our approach could be effectively applied to analyze similar scenarios. Furthermore, as recorded in endemic regions, future studies will highlight the transmission pathways moving from environmental sources to humans and subsequently to healthcare facilities.*

[Additional references]

- Kim SW, Park JW, Jung HD, Yang JS, Park YS, Lee C, Kim KM, Lee KJ, Kwon D, Hur YJ, Choi B. Risk factors for transmission of Middle East respiratory syndrome coronavirus infection during the 2015 outbreak in South Korea. Clinical Infectious Diseases. 2017 Mar 1;64(5):551-7.

**Comment #2**

The transmission also relies on the viral shedding in the infector and infectee; thus this factor should be reflected in the author's model.

**Answer #2**

In our study, the transmission rate incorporates both the number of contacts per unit time and the probability of infection following those contacts. This naturally includes viral shedding. We have accounted for host heterogeneity, which can vary based on factors such as distance from the infector and contact frequency, even if the shedding remains constant among infectors. To reflect this aspect, we have revised the text in section L88-93 as follows:

*Infectious hosts transmit the disease through viral shedding. We assumed that there is heterogeneity in the successful transmission based on contact frequency, duration, and distance between the infectee and infector, considering the types of hosts (HCWs, patients, and visitors) within the hospital setting.*

=============================================================================

**Comment #3**

I would strongly recommend the author read the article (PMID: 31961300) and incorporate the transmission dynamic in the author's model which primarily considers mathematics.

**Answer #3**

Thank you for recommending the paper. We added more introduction in L28-32 citing suggested paper and the other. In our study, we did not consider transmission through intermediate hosts, as our focus is on cases where infections are introduced to healthcare facilities in non-endemic regions, such as the Republic of Korea. We added a discussion on studies that describe the flow from environmental factors to humans and subsequently to hospitals, which has been observed in endemic regions (L257-259).

L28-32

*Studies conducted in endemic regions described the transmission pathways originating from environmental factors, progressing to human hosts, and ultimately reaching healthcare facilities. Our research focused on non-endemic regions where such intermediate host transmission may not play a significant role.*

L257-259

*Furthermore, as recorded in endemic regions, future studies will highlight the transmission pathways moving from environmental sources to humans and subsequently to healthcare facilities.*

**Reviewer summary:**

**Comment # 1**

The abstract as currently written does not have easily identifiable sections. The flow of content should be clear enough that the reader can identify the introduction, materials and methods, results and conclusion. For example, there is no introduction at all and the sentences can also be improved upon in general. Generally rewrite the abstract.

**Answer #1**

We revised the abstract section as follows for better readability and structure:

*Mathematical and statistical methods are invaluable in epidemiological investigations, enhancing our understanding of disease transmission dynamics and informing effective control measures. In this study, we presented a method to estimate transmissibility using patient-level data, with application to the 2015 MERS outbreak at Pyeongtaek St. Mary’s Hospital, the Republic of Korea. We developed a stochastic model based on individual case data to derive a likelihood function for disease transmission. Through scenario-based analysis, we explored transmission dynamics, including the role of superspreaders, and investigated how mask-wearing impacted infection control within the hospital. Our findings indicated that the superspreader during the MERS outbreak had approximately 25 times higher transmissibility compared to other patients. Under scenarios of prolonged hospital transmission periods, the number of cases could potentially increase threefold. The impact of mask-wearing in the hospital was significant, with reductions in the epidemic scale ranging from 17% to 77%, depending on the type of mask and intervention intensity. This study quantifies key risk factors in hospital-based transmission, demonstrating the effectiveness of intervention measures. The methodology developed here can be readily adapted to other infectious diseases, providing valuable insights for future outbreak preparedness and response strategies.*

**Comment #2**

There is a lot of copy-paste verbatim detected, moreover without citation. For example in lines 4-5 you write “The primary purpose of these investigations is to identify the occurrence of an outbreak or unusual increase in number of cases.”. This is the exact text as written in https://www.studysmarter.co.uk/explanations/medicine/epidemiology/outbreak-investigation/

**Answer #2**

We revised as follows:

*An epidemiological investigation is a systematic method used to determine the cause, source, and spread of a disease within a population, usually following an unusual increase in the number of cases. This process typically involves steps like verifying the outbreak, defining and identifying additional cases, using epidemiological methods to understand the disease transmission dynamics, and collecting data pertaining to cases, their characteristics, and potential risk factors.These data are directly utilized to estimate model parameters and simulate realistic outbreak patterns. This allows for evidence-based implementation of control measures to prevent further spread of the disease and reduce the risk of future outbreaks.*

=============================================================================

**Comment #3**

Improve clarity of most of your sentences. For example, in lines 7-8 you write “These data are then used to implement control measures to prevent further spread of the disease and to forestall future outbreaks.” However, it is not clear how data alone can be used to achieve that. Rather, the data is used to inform studies geared towards assessing the effectiveness of control measures.

**Answer #3**

We revised the text to enhance clarity as follows (L7-10):

*These data are directly utilized to estimate model parameters and simulate realistic outbreak patterns. This allows for evidence-based implementation of control measures to prevent further spread of the disease and reduce the risk of future outbreaks.*

**Comment #4**

In general, the flow of materials and methods needs to be heavily improved upon. For example, in lines 42-45, it is not clear how the two listed factors influence the probability of infection. I think the intended message is totally lost in the phrasing of the sentences.

**Answer #4**

The information from (1) is necessary to represent $N_{I}$ in Equation (1), and the information from (2) is required to set the total period applied in the likelihood function *L*. For clarity, the following two points have been added to the paper.

L58-62 (information (1))

*Let N indicate the total number of hosts. When assuming frequency-dependent transmission, ignoring natural birth and death, and fixing the number of infectious hosts as* $N_{I}$ *(estimated based on the first piece of information gathered from the epidemiological investigation mentioned above), the following equation is obtained:*

L83-85 (information (2))

*To apply this method to real data, the exact time range of exposure for each host is required, which is the second piece of information mentioned at the beginning of this subsection.*

=============================================================================

**Comment #5**

There is need to cite relevant literature throughout. For example, the derivations in equations (3)-(5) have already been published in several articles for example: 1) Dietz K and Schenzle D (1985) Proportionate mixing models for age-dependent infection transmission. Journal of Mathematical Biology 22, 117–120. 2) Becker NG (1989) Analysis of Infectious Disease Data. London: Chapman and Hall. And 3) Ssematimba A et al. (2018) Estimating the between-farm transmission rates for highly pathogenic avian influenza subtype H5N1 epidemics in Bangladesh between 2007 and 2013. Transboundary and Emerging Diseases 65, e127–e134. The authors need to acknowledge that.

**Answer #5**

We added the following references in the manuscript and cited in L68.

- Dietz K, Schenzle D. Proportionate mixing models for age-dependent infection transmission. Journal of mathematical biology. 1985 Jun;22:117-20.
- Becker NG. Analysis of infectious disease data. Chapman and Hall/CRC; 2017 Nov 22.
- Ssematimba A, Okike I, Ahmed GM, Yamage M, Boender GJ, Hagenaars TJ, Bett B. Estimating the between‐farm transmission rates for highly pathogenic avian influenza subtype H5N1 epidemics in Bangladesh between 2007 and 2013. Transboundary and emerging diseases. 2018 Feb;65(1):e127-34.

**Comment #6**

You are categorize events as Markovian and non-Markovian processes in the model events. This is based on the “memoryless property” of events. You may need to clarify/justify your choices for each event for the benefit of the readers.

**Answer #6**

We added clarification in the text regarding the categorization of events as Markovian and non-Markovian processes, explaining the rationale behind these choices for better reader understanding (L113-118):

*We categorized the events that could occur during an outbreak into delayed (non-Markovian) and nondelayed (Markovian). The event where a susceptible host is exposed to infection is assumed to be Markovian since it does not involve past points in time, while all other events are assumed to be non-Markovian as they are strongly influenced by past points in time. For example, the symptom onset after exposure is significantly affected by the past exposure point.*

========================================================================

**Comment #7**

In the abstract address the typographic error in “…by a maximum of 77Through…” There is also a missing “%” and minimum percentage.

**Answer #7:** Corrected

========================================================================

**Comment #8**

In line 17: You write “… age groups during COVID-19,…” add pandemic for completeness.

**Answer #8:** Corrected

========================================================================

**Comment #9**

Line 80: Use “indicating”

**Answer #9:** Corrected

**Comment #10**

There is a typo in Equation (6) change L_s to L_i in the second likelihood function.

**Answer #10:** Corrected

========================================================================

**Comment #11**

In line 99: mention the exact MATLAB function that you are referring to.

**Answer #11**

We used built-in function ‘fitdist’. We added the name of function in the manuscript (L123) as your suggestion.

========================================================================

**Comment #12**

In line 107, 123, 126, 153, 166 and elsewhere: spell out the “d” in “…PMH for 3 d…”?

**Answer #12**

We changed ‘d’ to ‘days’.

========================================================================

**Comment #13**

In the results section you use “Confidence Interval” and “Credibility Interval”. One is frequentist and the other is Bayesian. Be sure that you are rightly doing so based on the methods used in each case.

**Answer #13**

Thank you for your helpful comment. We set “confidence interval” for model simulation output (simulation runs) and “credible interval” for samples generated from Metropolis-Hastings algorithm.

========================================================================

**Comment #14**

Line 149: You write “…cases was 36.12, which was close to the actual number of cases…” For ease of comparison, mention here the actual number of cases.

**Answer #14**

We added the actual number using parenthesis in L173.

**Comment #15**

In line 188: what do you mean be “in order of”? Consider rephrasing the sentence for improved clarity.

**Answer #15**

We rephrased as follows (L210-211):

“The transmission rate induced by the superspreader was highest among patients, followed by visitors and then HCWs.”

========================================================================

**Comment #16**

Generally improve on your sentence to enhance the flow of content

**Answer #16**

Corrected. L2-8, L13-17, L28-42, L42-49, L88-93, L239-259
